# The FoxO3 Longevity Genotype Is Associated With Better Survival After Acute Myocardial Infarction in Older Patients

**DOI:** 10.1093/geroni/igaa057.474

**Published:** 2020-12-16

**Authors:** Randi Chen, Kazuma Nakagawa, Kamal Masaki, Tim Donlon, Ayako Elliott, Richard Allsopp, D Craig Willcox, Bradley Willcox

**Affiliations:** 1 Kuakini Medical Center, Honolulu, Hawaii, United States; 2 University of Hawaii, Honolulu, Hawaii, United States; 3 Okinawa International University,, Ginowan, Okinawa, Japan; 4 Kuakini Health Systems, Honolulu, Hawaii, United States

## Abstract

Background: Although numerous studies have been published on prognostic factors after a first acute myocardial infarction (AMI) among middle-aged men, little is known about the prognostic factors and genetic determinants for post-AMI elderly patients. Methods: The Kuakini Honolulu Heart Program (KHHP) is a prospective population-based study of cardiovascular disease (CVD) (defined as coronary heart disease (CHD) and stroke) among men of Japanese ancestry living in Hawaii. We identified 141 first AMI patients from participants of the KHHP exam 4 (1991-93) by ECG and/or cardiac enzymes (onset age: 73-96 years). Men who survived more than one year after AMI were followed for mortality from onset of AMI to December 2018 (all were deceased by 28 years of follow-up). All had FOXO3 genotype information available. Quantile regression was used to compare the median survival times of FOXO3-G allele carriers (longevity genotype) with FOXO3-TT homozygotes (common genotype). Results: Adjusting for age at AMI onset, baseline body mass index (BMI) and glucose levels (at exam 4), median survival times differed between FOXO3-G allele carriers and FOXO3-TT homozygotes by 2.1 years (95% CI=0.24-3.95, p=0.027). Adding other known CHD risk factors (i.e. hypertension, etc.) to the model reduced the difference to 2.03 years (95% CI=0.17-3.89, p=0.033). Furthermore, chi-square testing showed the mortality rate from CVD among FOXO3-G allele carriers was significantly lower than that among the common FOXO3-TT genotype. Conclusions: The data suggest that the FOXO3 longevity genotype reduces the risk of dying from CVD and extends survival time of elderly patients with acute myocardial infarction.

